# Direct Inferior Hip Dislocation With Intertrochanteric Fracture in an Adult: A Rare Case and Review of the Literature

**DOI:** 10.7759/cureus.94109

**Published:** 2025-10-08

**Authors:** Mark S Ayoub

**Affiliations:** 1 Department of Orthopaedics and Traumatology, University of California, San Francisco, Fresno, USA

**Keywords:** extracapsular hip fracture, intertrochanteric hip fracture, irreducible hip fracture-dislocation, orthopaedics and trauma, orthopaedic trauma surgery, traumatic hip dislocation, trochanter fracture with hip dislocation, unstable hip fracture

## Abstract

Direct inferior hip dislocation is the rarest type, sometimes referred to as luxatio erecta. To the author’s knowledge, an adult inferior hip dislocation with an associated displaced intertrochanteric fracture has only been reported once in the literature. Accordingly, there are few described treatment options for this injury. An 18-year-old male presented after a motor vehicle collision with an inferior hip dislocation and an associated ipsilateral intertrochanteric hip fracture. He was taken to the operating room urgently for the reduction of the dislocation and definitive treatment. In this case, a fracture table was utilized along with a Schanz pin to reduce the hip dislocation and for the reduction and intramedullary nailing of his intertrochanteric fracture.

## Introduction

Adult traumatic hip dislocations are most commonly posterior, with approximately 8-15% being anterior [1-3]. Anterior dislocations can be categorized as superior, pubic-type dislocations or inferior, obturator-type dislocations [4]. Even more rare, however, is the direct inferior hip dislocation, referred to as luxatio erecta, for which there have been only about 25 adult cases reported in the literature [5]. Of these, most are high-energy and occur without associated fractures of the hip [5]. To the author’s knowledge, the surgical treatment of an adult traumatic inferior hip dislocation with an associated intertrochanteric fracture has only been reported once in the literature [3], with one treatment method described. Presented is a combined inferior hip dislocation with an intertrochanteric fracture, managed with fracture table-assisted reduction of both injuries, followed by intramedullary nail fixation - an approach not previously described to the author’s knowledge.

## Case presentation

**Figure 1:**
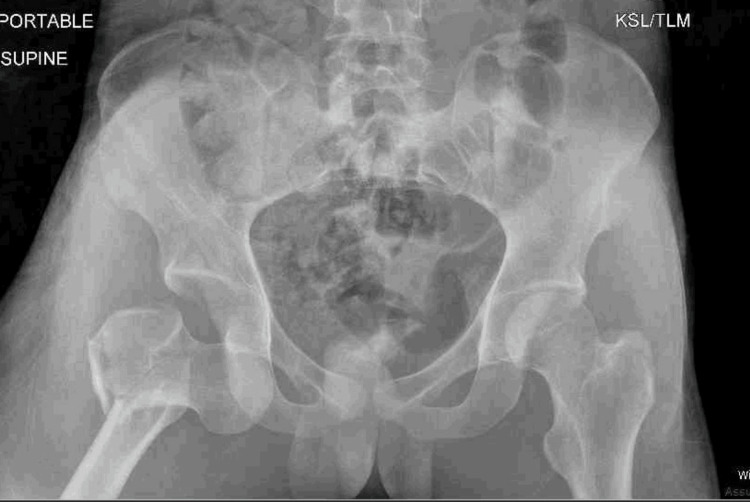
Anteroposterior (AP) radiograph showing direct inferior hip dislocation with an associated intertrochanteric fracture The obturator ring remains unobstructed in contrast with an anterior-obturator dislocation.

**Figure 2:**
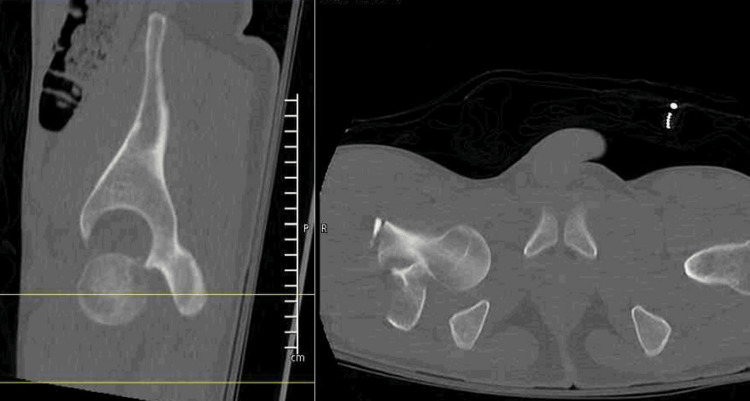
Sagittal and corresponding axial CT image of the right hip showing inferior hip dislocation The femoral head sits directly inferior to the acetabular lip without anterior or posterior displacement.

**Figure 3:**
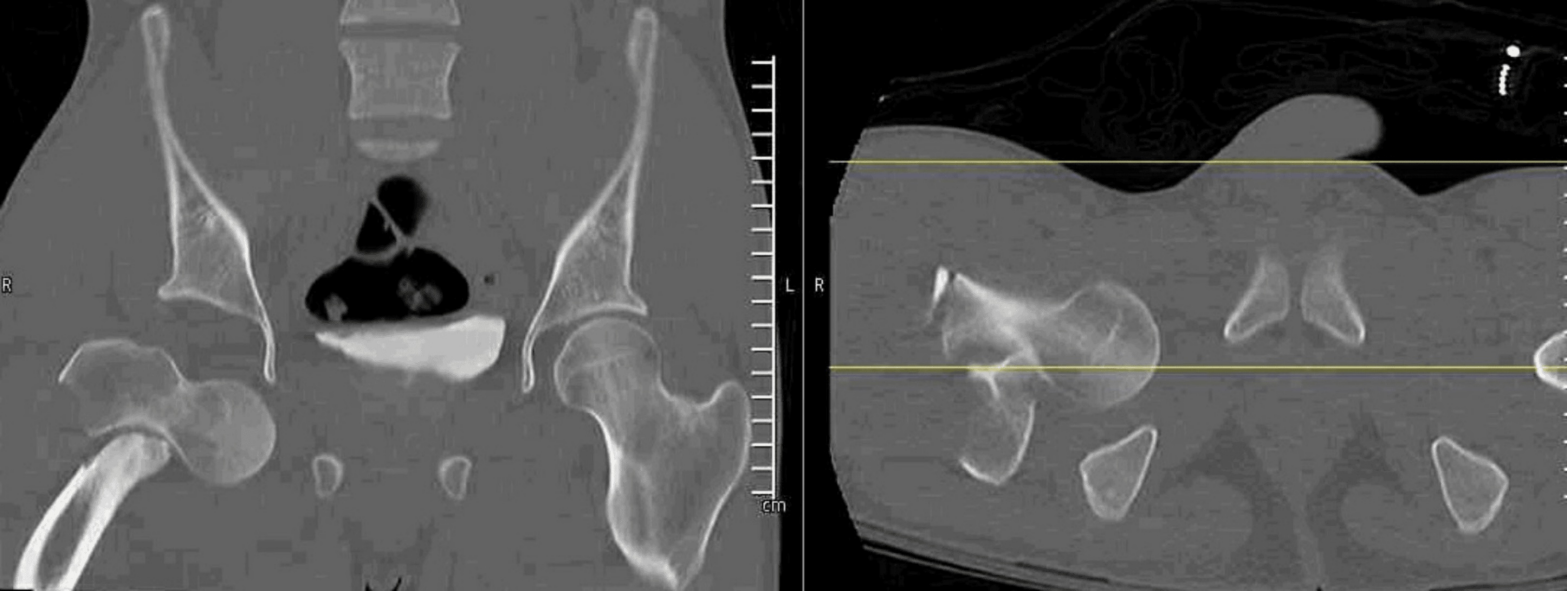
Coronal and corresponding axial CT images showing an ipsilateral intertrochanteric hip fracture associated with a direct inferior hip dislocation.

An 18-year-old male presented to an outside hospital as an unrestrained front-seat passenger in a head-on motor vehicle collision. He was transferred nine hours after injury with a right-sided inferior hip dislocation and an associated ipsilateral intertrochanteric hip fracture. On physical examination, his right lower extremity was shortened approximately 4 cm and externally rotated 30 degrees. He remained neurovascularly intact with full sciatic nerve strength and sensation noted. He had significant pain with the range of motion of the extremity. There was no skin compromise or ecchymosis noted on the extremity. Vital signs were stable. Radiographic imaging was obtained (Figure 1) showing a direct inferior hip dislocation with associated intertrochanteric fracture. A CT scan was obtained, confirming the direction of the dislocation with no evidence of femoral head impaction, intra-articular loose bodies, or associated hip or pelvic ring injuries (Figures 2, 3). His other associated injuries included conservatively treated fractures of the sternum, ribs, and clavicle. The Injury Severity Score (ISS) was calculated to be 22.

**Figure 4:**
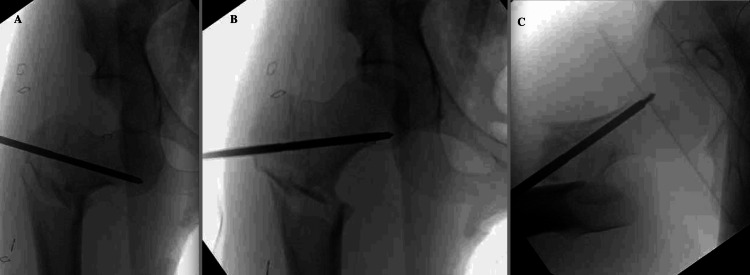
Reduction maneuver of inferior hip dislocation prior to fracture reduction utilizing fracture table-assisted traction and Schanz pin A. A 5.0 mm Schanz pin was angled superior to inferior into the proximal segment. B. Reduction was achieved in one smooth attempt by levering the pin inferiorly and by using a gentle upward directed force. Traction was applied to the limb prior to reduction to disimpact the femoral shaft. C. Lateral fluoroscopic imaging showing anteriorly placed pin to avoid the femoral head blood supply.

He was taken to the operating room (OR) urgently, within 2.5 hours, prior to any reduction attempts, and positioned supine on a fracture table. Traction was applied through the boot assembly to disengage the femoral shaft, and a 5.0 mm Schanz pin was placed into the femoral neck under fluoroscopic guidance. The pin was used to guide the femoral head into the acetabulum with one attempt (Figure 4). Reduction was performed approximately 12 hours after the time of injury. 

**Figure 5:**
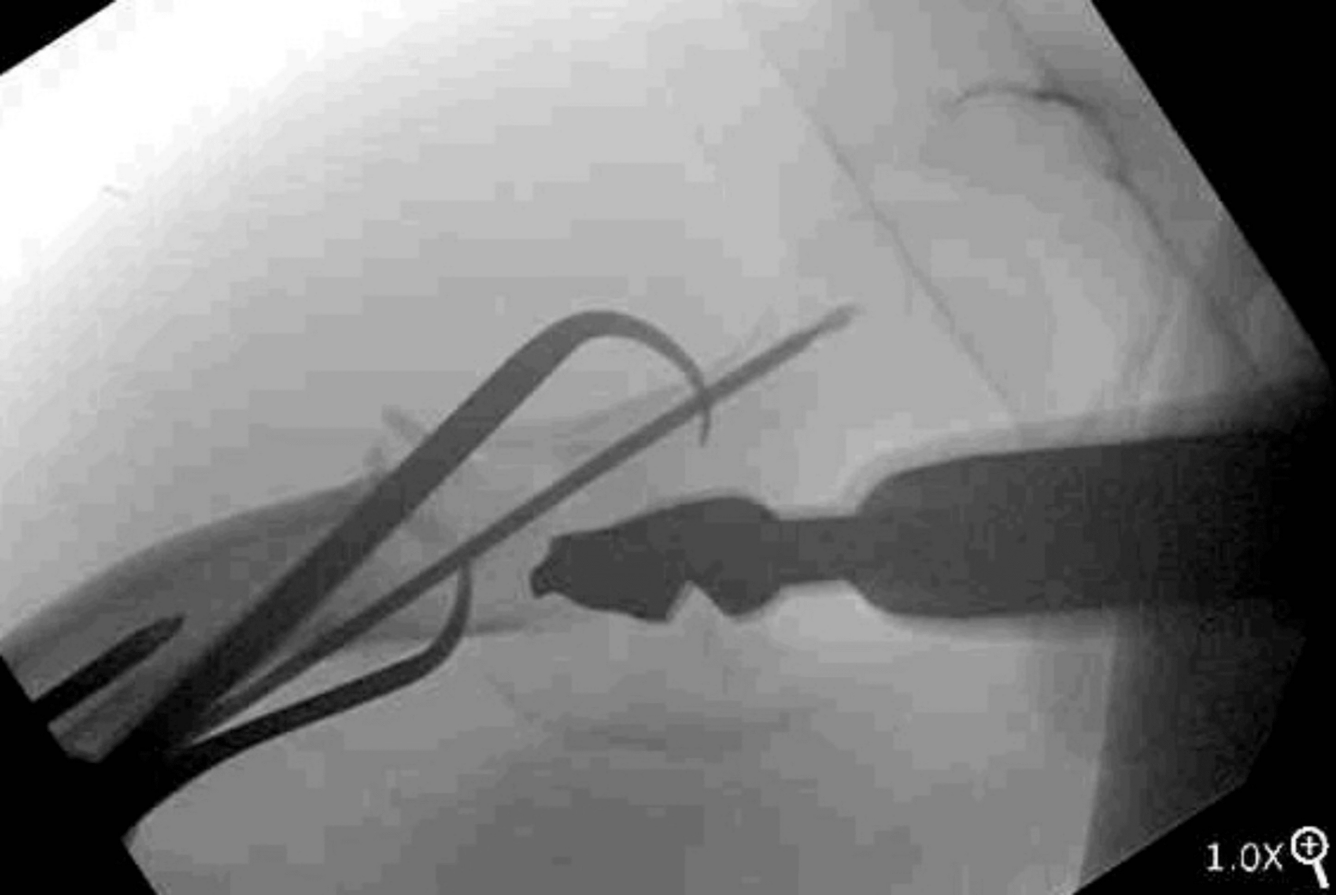
Reduction maneuver for intertrochanteric fracture after reduction of the inferior hip dislocation The sagittal plane deformity was addressed using a bone hook to control the proximal segment, a unicortical Schanz pin to elevate the distal segment, and an anteriorly placed wire to provisionally hold the reduction.

**Figure 6:**
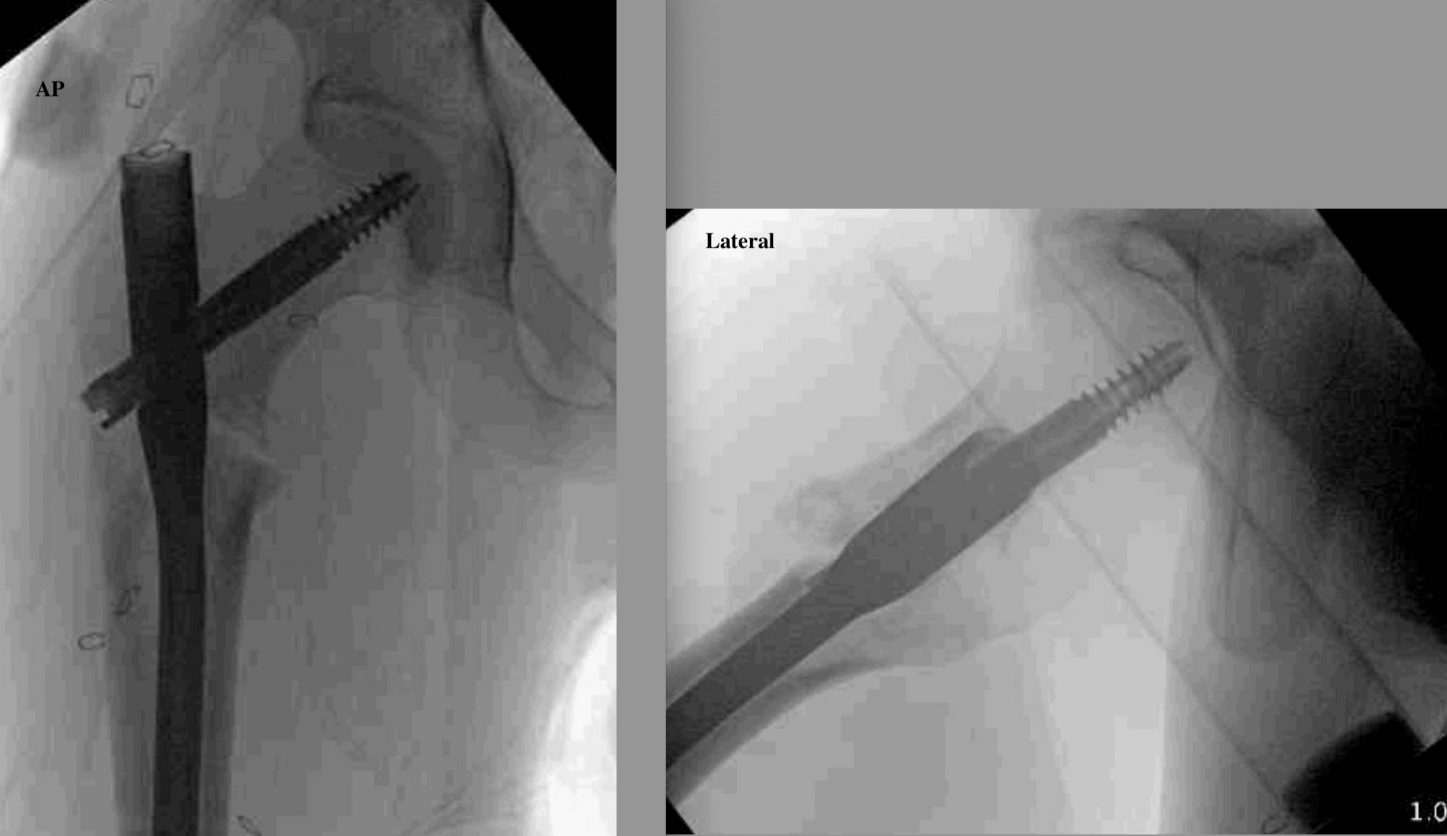
Anteroposterior (AP) and lateral fluoroscopic images showing final reduction and intramedullary nail fixation of direct inferior hip dislocation and intertrochanteric fracture Slight apex anterior deformity is noted on the lateral image.

The intertrochanteric fracture was then addressed. The previously applied traction had restored the length, while the initial Schanz pin was used to joystick the proximal segment. A bone hook was applied to the proximal segment anteriorly, and a second unicortical Schanz pin was placed in the shaft to elevate the distal segment and restore sagittal alignment (Figure 5). A retrograde 2.8 mm guidewire held the reduction, anterior to the planned nail path. Definitive fixation was achieved with a cephalomedullary nail using a medialized trochanteric start point to avoid varus deformity (Figure 6); the nail was distally locked dynamically to allow for additional compression at the fracture site. Of note, there was some residual apex anterior deformity that was seen on final imaging and accepted by the treating surgeon. In addition, despite the slightly posterior placement of the lag screw on the lateral view, there was excellent purchase, and accordingly, it was left in place. The hip was gently ranged at the conclusion of the procedure, and there was no clinical instability detected. Surgical time was noted to be 141 minutes, and the estimated blood loss was 200 cc. 

**Figure 7:**
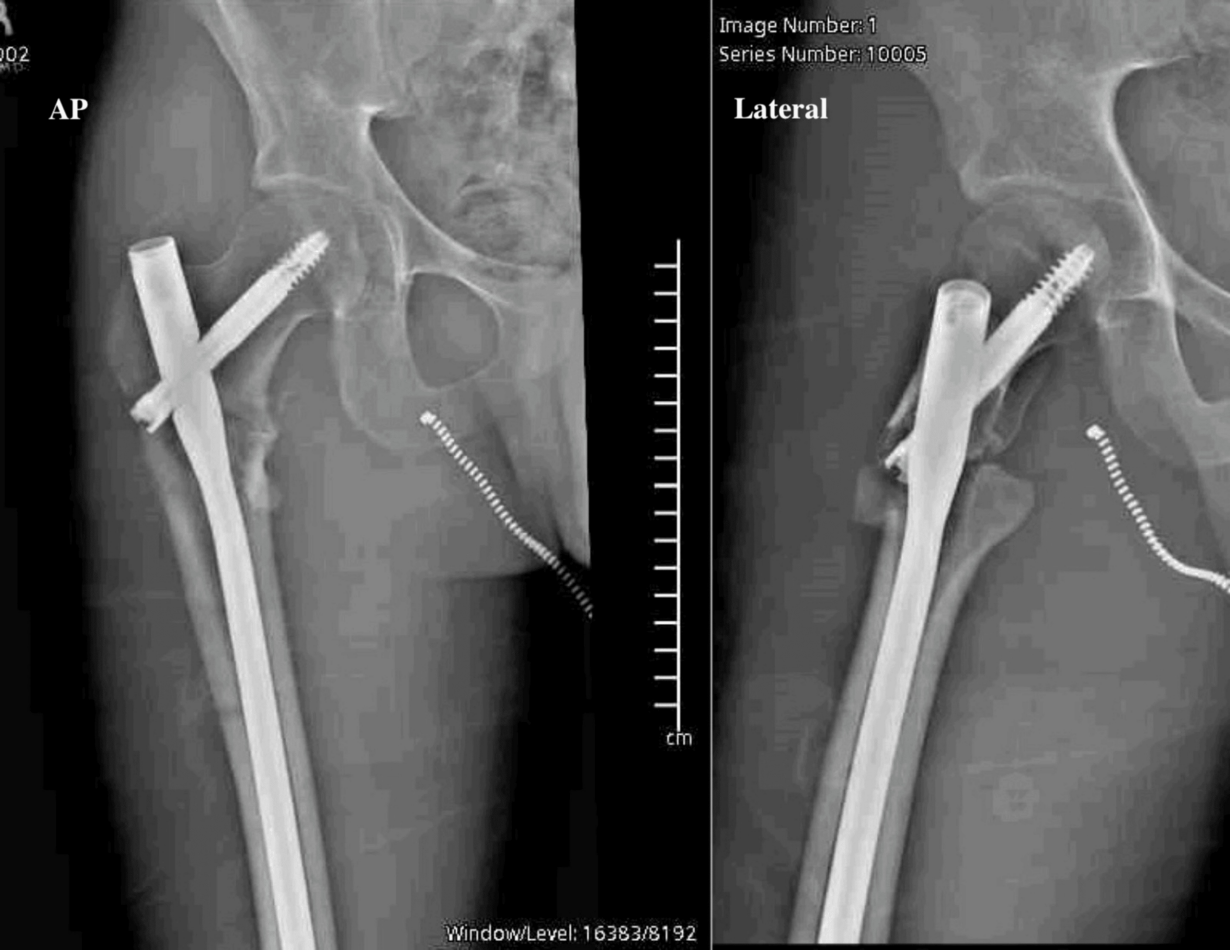
Anteroposterior (AP) and lateral radiographs of right hip eight weeks postoperatively showing concentrically reduced hip joint and acceptable fracture reduction after closed reduction of inferior hip dislocation and closed reduction and intramedullary nailing of associated intertrochanteric fracture

Postoperatively, he was made non-weight-bearing on the right lower extremity with no hip precautions. He presented for follow-up at eight weeks (Figure 7). He was neurovascularly intact, all incisions were healed, and leg lengths were grossly equal. His hip had a near full range of motion on exam with approximately 110 degrees of flexion, 30 degrees of internal and external rotation, and no apprehension or evidence of instability. He had minimal pain at that time, weight bearing was advanced, and he was subsequently lost to follow-up despite multiple attempts to contact him at five, 12, and 14 months postoperatively.

## Discussion

True inferior hip dislocations are exceedingly rare. While some authors have classified anterior-obturator dislocations as inferior [5,6], several have emphasized that these are distinct entities. In true direct inferior dislocations, the femoral head lies directly inferior to the acetabular rim rather than anterior, and the obturator ring remains unobscured, features that distinguish them from the anterior-obturator variants [3,7,8]. The distinction is clinically relevant as each requires a different reduction maneuver. Anterior-obturator dislocations can be reduced using hip flexion, adduction, and external rotation to unlock the head from the obturator foramen, followed by axial traction [9]. By contrast, true inferior dislocations are reduced using axial traction, extending the thigh, and applying additional internal rotation [10].

As with other hip dislocations, inferior dislocations are usually high-energy injuries that require significant force [5]. There are two reported mechanisms. Mechanism 1 is a direct impact on a flexed knee and hip, creating a sub-ischial type of dislocation [11]. Mechanism 2 is a direct force to an abducted hip, leading to further abduction and flexion and external rotation, which lever the femoral head out of the capsule [11]. This could explain this patient’s mechanism of injury: he sustained a high-velocity frontal impact with the hip flexed and abducted, likely driving the femoral head inferiorly beneath the acetabulum. Patients with inferior hip dislocations without an associated peritrochanteric fracture present with the femur parallel to the axis of the body and torso and in neutral rotation; this is a distinct clinical appearance that should cue the provider to consider a direct inferior hip dislocation as opposed to a hip fracture or anterior dislocation variant. Almost all isolated adult inferior dislocations can be treated with closed reduction [5,12], although open reduction has been reported in select cases [7]. 

Femoral head impaction has been described with atypical hip dislocations [3,7,13,14]. Accordingly, with inferior dislocations, CT imaging should be analyzed to assess for this preoperatively, and impaction should be addressed if needed. Prompt reduction is recommended to minimize the risk of avascular necrosis in inferior dislocations [3,5,15], although this is based on data extrapolated from posterior hip dislocations. With associated fractures, particularly peritrochanteric or femoral shaft fractures, the reduction often is not feasible in the emergency room, requiring direct manipulation of the head-neck segment in the OR [3]. 

Associated femoral shaft [10,16] and pubic rami fractures [12] have been reported with inferior dislocations. In addition, Yang et. al described an iatrogenic greater trochanter fracture during a closed reduction attempt requiring open reduction internal fixation [17]. 

A possible mechanism for an associated peritrochanteric fracture with luxation erecta is the collision of the greater trochanter with the iliac bone [18,19]. Because of the associated intertrochanteric fracture, the limb can be found in a neutral, rather than hyperflexed position, and with shortening and external rotation similar to a traditional hip fracture [3]. 

A surgically treated adult inferior hip dislocation with associated intertrochanteric hip fracture has only been reported once in the literature [3]. Singh et al. (2006) described a 36-year-old male involved in an accident, thrown from the top of a motor vehicle. The reduction of the hip dislocation was performed in the OR supine without extremity traction, using a Schanz pin to reduce the femoral head, and definitive fixation of the fracture was performed with a dynamic hip screw [3]. He was placed in Buck’s traction for two weeks postoperatively, advanced to toe-touch weight bearing until eight weeks, and then allowed to weight bear as tolerated. At 2.5-year follow-up, they had no signs of avascular necrosis and had symmetric hip motion. 

An alternative case and treatment are presented here in an 18-year-old male involved in a motor vehicle collision. While Schanz pin-assisted reduction was also utilized, treatment differed with fracture table, traction-assisted reduction of this hip dislocation, and intramedullary nail fixation of the intertrochanteric fracture rather than the dynamic hip screw. Contrary to the prior report, this patient was not placed into traction postoperatively; however, intraoperative stability testing for the hip joint should be performed if there are any concerns about hip instability. Weight bearing was advanced at a similar time frame for both patients, eight weeks postoperatively. Both this and the prior report emphasized early reduction to lower the risk of avascular necrosis [3]. Finally, while the prior study did not include lateral imaging after fixation, in this patient, there was a slight apex anterior angulation after final implant placement that was deemed acceptable by the treating surgeon due to the extracapsular nature of the fracture. Surgeons should certainly consider open reduction and cerclage cabling if needed to improve the reduction and weigh this against the risks of potentially devascularizing the fracture, increasing blood loss, and increasing anesthetic time. Table 1 summarizes all reported true inferior hip dislocations associated with fractures. 

**Table 1:** Literature review of treatment and outcomes of associated fractures with true inferior hip dislocations ORIF, open reduction internal fixation; IMN, intramedullary nail; MVC, motor vehicle collision; AVN, avascular necrosis; GT, greater trochanteric; HO, heterotopic ossification; AROM, active range of motion

Study, year	Age, mechanism	Associated fracture	Treatment	Outcome
Singh et al. [3], 2006	36 y/o M- MVC	Intertrochanteric hip	Closed reduction, ORIF with screw and side plate	2.5 years: full AROM, (-)AVN
Yang et al. [17], 2014	47 y/o M- motorcycle accident	Iatrogenic GT fracture during Closed Reduction	ORIF GT fracture with screw fixation, three weeks of bed rest	2 years: pain-free hip joint, Full AROM
Jain et al. [10], 2015	17 y/o M- MVC	Femoral shaft	Closed reduction, femur IMN	4 years: (+)HO, (-)AVN
Moussa et al. [20], 2016	24 y/o M -MVC	Nondisplaced femoral head, nondisplaced intertrochanteric	Closed reduction, closed treatment (6 weeks NWB)	3 months: full weight bearing
Syam et al. [12], 2017	38 y/o M-MVC	Bilateral pubic rami, iliac wing fracture	Closed reduction, closed treatment	_
Dharmshaktu et al. [16], 2020	18 y/o M- MVC	Femoral shaft	Closed reduction, femur IMN	9 months: (-)AVN, pain-free ambulation

Contrary to prior reports, the authors note that intraoperative traction, whether achieved manually or through the use of an OR table, may facilitate the reduction of the dislocation, as it disengages the femoral shaft, allowing the proximal head-neck segment to be mobilized and reduced more easily. Moreover, the use of fracture table traction provided controlled restoration of length, minimized the need for multiple assistants, and facilitated the reduction of both the hip dislocation and the intertrochanteric fracture. 

Intertrochanteric hip fractures can be treated using either radiolucent or fracture tables. Radiolucent tables may require additional assistants to apply traction but can be helpful for patients with morbid obesity or polytrauma patients requiring multiple procedures [21]. Conversely, fracture tables may be challenging with obese patients and carry a risk of perineal injury but are still used by many surgeons for familiarity and the reduced need for assistants [21]. For those who prefer the fracture table, it remains a viable option for managing both the fracture and dislocation components of this injury.

Finally, the strategic placement of the proximal segment Schanz pin can be beneficial. A Schanz pin provides multiplanar control of the femoral head to more easily guide it into the acetabulum, as well as direct control of the proximal segment when reducing the fracture. The pin can be aimed superior to inferior to assist with the dislocation reduction maneuver and can be placed anteroinferiorly along the femoral neck to preserve femoral head blood supply while remaining outside the planned path for the final implant. 

This report is limited by the fact that the patient was lost to follow-up after eight weeks despite multiple attempts to contact him. As a result, long-term functional and radiographic outcomes could not be assessed. Nonetheless, the case contributes to the limited literature by documenting the injury pattern, reduction technique, and short-term recovery, while reinforcing the distinction of true inferior hip dislocations as a separate entity from anterior-obturator variants.

## Conclusions

A true direct inferior hip dislocation with associated intertrochanteric hip fracture is an extremely rare injury pattern. An alternative treatment strategy involves using fracture table-assisted reduction of both the hip dislocation and intertrochanteric fracture, and intramedullary nail fixation of the fracture. Surgeons who are more comfortable treating an intertrochanteric hip fracture on a fracture table can use the same setup for the reduction of this hip dislocation. In addition, strategic Schanz pin placement in the proximal segment can facilitate the hip dislocation reduction maneuver while allowing the pin to be used during peritrochanteric fracture treatment without obstructing the final implant. This injury requires urgent surgical intervention, as closed reduction in the emergency room is not feasible, and avascular necrosis may be a significant risk with delayed reduction, as it is for other types of hip dislocations. Finally, this is a single case report with only eight weeks of follow-up; findings should be interpreted with caution.
